# Semi-Annual Meeting of the Esculapian Society, Held in Marshall, Ill.

**Published:** 1858-07

**Authors:** C. M. Hamilton, D. W. Stormont

**Affiliations:** President; Secretary


### PROCEEDINGS OF MEDICAL SOCIETIES.

### SEMI-ANNUAL MEETING OF THE ESCULAPIAN SOCIETY, HELD IN MARSHALL, ILLINOIS, MAY 26TH AND 27TH, 1858.

The society met, May 26th, in the office of Dr. Payne, and was called to order at 11 o'clock. The minutes of the last meeting were read by the secretary, and approved. Drs. Duncan and Mitchell were appointed to fill vacancies in the board of censors.

Drs A. K. Spears and S. C. Hoge, of Kansas, Edgar Co., were elected members of the society.

On motion, adjourned to meet in the Court House at 1 o'clock.

### AFTERNOON SESSION.

The society met, as per adjournment, the president, Dr. Hamilton, in the chair.

Dr. Hoge read a thesis on Cynanche Trachealis, which was discussed by Drs. H. R. Payne, Duncan, Mitchell, McCord, Johnson and Hamilton.

Dr. Stormont read a paper on Typhoid Pneumonia. Discussed by Drs. H. R. Payne, Johnson, Mitchell, Herrick, F. R. Payne, Steele, Duncan and McCord.

Dr. Bowers reported a case of Chorea, which was discussed by several members.

Drs. Johnson, F. R. Payne and Stormont were appointed a busiuess committee, to report to-morrow morning.

Dr. McCord's resolution, offered at the last meeting, in reference to publishing the transactions of this society, was taken up. On motion of Dr. Stormont, it was indefinitely postponed.

Adjourned to meet at 8 o'clock.

### EVENING SESSION.

The society met, according to adjournment, the president in the chair.

Dr. Herrick being introduced, delivered an excellent address, to a large audience, on the Advancement of Medical Science.

On motion of Dr. Payne, the thanks of the society were tendered Dr. Herrick for his address, and a copy requested for publication.

On motion of Dr. Johnson, the society adjourned, to meet tomorrow morning, 8 o'clock.

MAY 27TH.—MORNING SESSION.

The society met, as per adjournment.

Dr. McCord read an essay on Croup, which was discussed by Drs. Hinkle, H. R. Payne, Stormont, Johnson and McCord.

Dr. Mitchell reported a case of Complicated Labor.

Dr. Hinkle read a paper on Some of the Causes which have produced a Change in Medical Practice. Discussed by Drs. Johnson, York, Davis and Hinkle.

Dr. Duncan reported a case of Neuralgia. Discussed by Drs. Hinkle, Steele, York, Mitchell and Payne.

Dr. Davis read an essay on Gonorrhea. Discussed by Drs. Hinkle, Spears, York and Johnson.

Dr. Bowers read an essay on the Sequences of Intermittent Fever. Discussed by Drs. York, Bowers, Hinkle and Gorham.

The business committee reported the following subjects, and the gentlemen whose names are opposite were appointed to write thereon for the next meeting, viz:

Effects of Malaria in Modifying Disease—Dr. Payne.
Uterine Tumors—Dr. Chambers.
Diptheritis—Dr. Davis.
Delirium Tremens—Dr. Gorham.
Epilepsy—Dr. F. R. Payne.
Milk-Sickness—Dr. McAllister.
Ophthalmia—Dr. Spears.
Menorrhagia—Dr. Mitchell.
Appearance of the Tongue as an Indication of Disease—Dr. Herrick.
Vomiting in Pregnancy—Dr. TenBroek.
Croup—Dr. Stevens.
Hemorrhoids—Dr. Hoge.
Pneumonia—Dr. VanDyke.
Chronic Cystitis—Dr. Johnson.
Dropsical Effusions—Dr. Bridges.
Typhoid Fever—Dr. Bowers.

Neuralgia—Dr. Duncan.

The Physiological and Therapeutical Effects of Chlorate of Potass—Dr. Hinkle.

Chemico-Medical Combinations—Dr. York.

Dr. S. York was appointed to deliver the next public address.

On motion, adjourned to meet at 1½ o'clock.

AFTERNOON SESSION.

The society met according to adjournment, the president in the chair.

Dr. York reported a case of poisoning by tinct. stramonium.

York was selected as the place for holding the next meeting.

Drs. McCord, Johnson and Gorham were appointed the committee of arrangements.

On motion of Dr. Herrick, the following resolution was adopted, viz:

*Resolved*, That the thanks of this society be tendered the members of the society who reside in Clark County, for their hospitality to its members during their stay in Marshall, and especially for the sumptuous supper which they furnished.

The following resolution, offered by Dr. York, was adopted, viz:

*Resolved*, That this society is of opinion that the longer continuance of the practice of giving public suppers is inexpedient and ought to be discontinued.

Dr. Stormont offered the following resolution, which was adopted, viz:

*Resolved*, That a committee of three be appointed, whose duty it shall be to devise means to secure the enactment, by our next Legislature, of a law for registering the births, deaths and marriages in this State; said committee to report at the next meeting of this society.

Drs. Stormont, F. R. Payne and T. D. Washburn were appointed.

It was ordered that a copy of these proceedings be furnished the *Chicago Medical Journal* for publication.

On motion, adjourned to meet in York the last Wednesday in October.

C. M. HAMILTON, *President*.

D. W. STORMONT, *Secretary*.

